# Scoping review of regulatory transparency in AI-based radiology software: analysis of PMDA-approved SaMD products

**DOI:** 10.1007/s11604-025-01942-y

**Published:** 2026-01-14

**Authors:** Tomohiro Kikuchi, Shannon L. Walston, Hirotaka Takita, Yasuhito Mitsuyama, Rintaro Ito, Masahiro Hashimoto, Takeshi Nakaura, Hiroaki Hyakutake, Sho Kawabe, Harushi Mori, Daiju Ueda

**Affiliations:** 1 Department of Radiology, Jichi Medical University, 3311-1, Yakushiji, Shimotsuke-shi, Tochigi, 329-0498 Japan; 2 Medical AI Promotion Institute Co., Ltd., Life Science Building, 12-9 Nihonbashi Odemmachi, Chuo-ku, Tokyo, Japan; 3 Department of Artificial Intelligence, Graduate School of Medicine, Osaka Metropolitan University, Asahi-machi, Abeno-ku, Osaka, 545-8585 Japan; 4 Department of Diagnostic and Interventional Radiology, Graduate School of Medicine, Osaka Metropolitan University, 1-4-3 Asahi-machi, Abeno-ku, Osaka, 545-8585 Japan; 5 Smart Data and Knowledge Services Department, German Research Center for Artificial Intelligence (DFKI GmbH), 67663 Kaiserslautern, Germany; 6 Department of Innovative BioMedical Visualization, Nagoya University Graduate School of Medicine, Showa-ku, Nagoya, Japan; 7 Department of Radiology, Keio University School of Medicine, Shinjukuku, Tokyo Japan; 8 Department of Diagnostic Radiology, Kumamoto University Graduate School of Medicine, Chuo-ku, Kumamoto, Japan; 9 Center for Health Science Innovation, Osaka Metropolitan University, Asahi-machi, Abeno-ku, Osaka, 545-8585 Japan

**Keywords:** Artificial intelligence, Software as a medical device, Radiology, PMDA, Scoping review, Data transparency

## Abstract

**Background:**

The integration of artificial intelligence (AI) in radiology has accelerated globally, with Japan’s Pharmaceuticals and Medical Devices Agency (PMDA) approving numerous AI-based Software as a Medical Device (SaMD) products. However, the transparency and completeness of clinical evidence available to healthcare providers remain unclear.

**Purpose:**

To systematically evaluate the availability and transparency of clinical evidence in package inserts of PMDA-approved AI-based radiology SaMD products, identifying gaps that may impact clinical implementation.

**Materials and methods:**

We conducted a scoping review of all PMDA-approved SaMD products as of December 31, 2024. Products were included if they utilized AI technology and were classified for radiology applications. Data extraction focused on product characteristics, study designs, demographic information, and performance metrics.

**Results:**

Of 151 approved SaMD products, 40 utilized AI technology, with 20 specifically designed for radiology applications. Critical gaps were identified in demographic reporting, with no products providing complete case demographic data. Performance metrics varied widely, with sensitivity ranging from 67.7% to 100% in standalone studies. Physician-assisted studies consistently demonstrated performance improvements but lacked stratified results by characteristics in all cases.

**Conclusion:**

Current package insert requirements provide insufficient transparency for evidence-based clinical implementation of AI-based radiology SaMD. Enhanced regulatory frameworks and industry-led initiatives for comprehensive validation are essential for safe and effective AI deployment in Japanese healthcare.

## Introduction

The rapid advancement of artificial intelligence (AI) technologies has fundamentally transformed the landscape of medical imaging and radiology practice worldwide [1]. In Japan, the Pharmaceuticals and Medical Devices Agency (PMDA) has established itself as a pioneering regulatory body in the approval of AI-based Software as a Medical Device (SaMD) products, with a notable concentration in radiology applications [2]. As of 2024, Japan is an early adopter of AI‑based medical devices, with a dedicated SaMD review office and dozens of approved products, reflecting its technologically advanced healthcare system and progressive regulatory framework [3].

The integration of AI into clinical radiology practice presents unique challenges that extend beyond traditional medical device implementation. Unlike conventional imaging equipment, AI-based software systems exhibit performance characteristics that can vary significantly based on patient demographics, imaging protocols, and institutional practices [4]. This variability necessitates a higher standard of transparency in clinical evidence to enable healthcare providers to make informed decisions about implementation and to understand the limitations of these technologies in their specific clinical contexts [5].

Package inserts, known as “tenpu-bunsho” in Japanese regulatory terminology, serve as the primary source of product information for clinicians and healthcare institutions [6]. These documents are legally required to contain essential information about the safety, efficacy, and proper use of medical devices [7]. For SaMD products that utilize AI technologies (hereinafter referred to as “AI-based SaMD”), package inserts represent a critical communication channel between manufacturers, regulators, and end-users, theoretically providing the evidence base necessary for safe and effective clinical implementation. However, the rapid pace of AI development and deployment has outstripped the evolution of regulatory documentation standards. International studies have highlighted significant variations in the quality and completeness of clinical evidence provided for AI-based medical devices, with particular concerns about the generalizability of performance claims across diverse patient populations [8, 9]. The unique characteristics of the Japanese healthcare system, including its demographic composition and clinical practice patterns, make it particularly important to understand how well current regulatory documentation serves the needs of Japanese healthcare providers [10].

Furthermore, the implementation of AI in radiology intersects with Japan’s evolving framework for medical data governance, including the Next Generation Medical Infrastructure Act and the Personal Information Protection Act [10]. These regulations create both opportunities and challenges for the development and validation of AI systems, requiring specialized expertise to navigate the complex landscape of data privacy, clinical validation, and regulatory compliance.

The objective of this study is to systematically evaluate the transparency and completeness of clinical evidence provided in package inserts for PMDA-approved AI-based radiology SaMD products. While technical details absent from package inserts may occasionally be found in manufacturer white papers or academic publications, this study specifically focuses on package inserts. This is because package inserts serve as the most immediate, accessible, and authoritative first point of reference for clinicians when implementing or using these applications. By analyzing the availability of demographic data, performance metrics, and study design information, we aim to identify gaps that may impact clinical implementation and to provide recommendations for enhancing regulatory transparency in this rapidly evolving field.

## Materials and methods

### Data source and search strategy

This study was designed as a scoping review and reported in accordance with the Preferred Reporting Items for Systematic reviews and Meta-Analyses extension for Scoping Reviews (PRISMA-ScR) [11]. The review protocol was not prospectively registered. On 31 May 2025 we downloaded the Excel spreadsheet listing all PMDA‑approved SaMD products from the PMDA website [12]. We filtered the data to include only records approved on or before 31 December 2024. Two investigators independently applied these filters, then compared outputs and confirmed complete concordance.

### Inclusion and exclusion criteria

Products were included in the analysis if they met the following criteria: (1) approved by PMDA as SaMD, (2) designated as “AI-utilizing medical devices” in the official PMDA approval list, and (3) indicated for use in radiology applications, including but not limited to X-ray, computed tomography (CT), magnetic resonance imaging (MRI), and ultrasound imaging. Specifically regarding criterion (2), the PMDA defines products in this category as SaMD possessing functions utilizing AI technology intended to improve patient outcomes, excluding those solely for workload reduction [12]. Products were excluded if they were (1) non‑AI software; (2) applications dedicated to endoscopy or laparoscopy; (3) software intended for pathology, other specimen‑based laboratory testing, or electrocardiography—applications not directly related to radiology; or (4) consumer‑oriented wearable products such as Apple Watch applications. This exclusion criteria ensured focus on traditional radiology imaging modalities.

### Data extraction framework

The extraction framework encompassed the following categories: Product characteristics included approval ID, version number, product name and overview, imaging modality, marketing authorization holder, manufacturer, and study design used for regulatory approval. For each product, we documented whether approval was based on standalone performance testing, physician-assisted studies, or both.

Clinical study characteristics were extracted in detail, including total sample size, number of positive cases, patient demographic information (nationality, ethnicity, race, sex, age distribution), imaging equipment vendors, and participating clinical sites. We specifically noted whether demographic breakdowns were provided and whether performance metrics were stratified by these characteristics.

Performance metrics extraction focused on standard diagnostic accuracy measures including sensitivity, specificity, positive predictive value (PPV), negative predictive value (NPV), area under the receiver operating characteristic curve (AUC), and false positive rates. We considered performance metrics to be “reported” if at least one quantitative measure of diagnostic performance was provided, without restricting the requirement to specific metrics (e.g., sensitivity or AUC) to accommodate diverse study designs. For physician-assisted studies, we additionally extracted information about reader characteristics, including number of readers, specialization, experience levels, and geographic distribution.

### Data analysis

Descriptive statistics were used to quantify reporting completeness. We defined “complete demographic data” as the reporting of all three key characteristics: age distribution, sex distribution, and nationality, ethnicity, or race. For every product we recorded the presence (coded 1) or absence (coded 0) of the following transparency items: total case number; demographic breakdowns for nationality/ethnicity/race, sex, and age; vendor‑specific information; site‑specific information; and performance metrics stratified by any demographic or technical variable. We calculated the frequency and percentage of products reporting each transparency item. We did not calculate composite quality scores or rank the products, as the primary objective was to assess the presence or absence of specific information.

Performance metrics were summarized using ranges and medians where appropriate. For products reporting multiple performance values (e.g., at different thresholds or for different findings), we documented the manufacturer‑designated primary metric. Physician-assisted studies were analyzed separately to assess the impact of AI assistance on diagnostic performance.

## Results

### Product landscape and eligibility

**Fig. 1:**
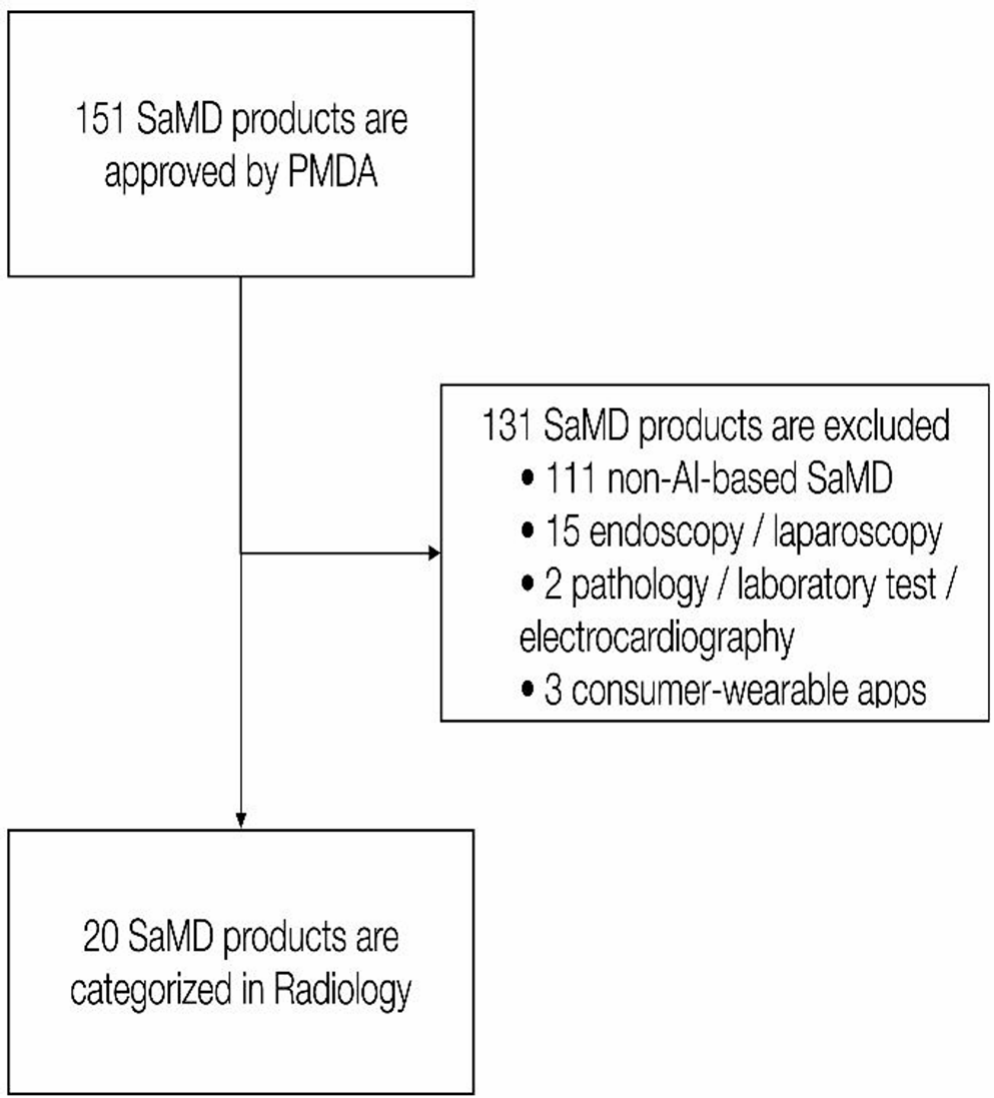
Eligibility. The flowchart illustrates the screening process for identifying eligible AI-based radiology SaMD products. Starting from all SaMD products approved by the PMDA, products were excluded based on pre-defined criteria, resulting in the final cohort for analysis. *SaMD, software as a medical device; PMDA, pharmaceuticals and medical devices agency; AI, artificial intelligence

The PMDA data listed 151 SaMD approvals. Of these, 111 products were non‑AI and excluded (exclusion criteria 1). Among the remaining 40 AI‑based SaMD products, 15 were endoscopy/laparoscopy applications (exclusion criteria 2), two were pathology/laboratory/electrocardiography applications (exclusion criteria 3), and three were consumer‑oriented wearable apps (exclusion criteria 4). Consequently, 20 AI‑based radiology products met all eligibility criteria and were included in the analysis (Fig. 1). Twelve products had undergone at least one package‑insert revision, and our analysis evaluated the latest version available as of June 2025.

**Table 1:** Product characteristics

Citation	Approved ID	Version	Product overview	Modality	Marketing authorization holder	Manufacturer	Study design for approval
13	30200BZX00202000	2nd ed., Apr 2022	Analyzes chest CT scans to help doctors detect and mark potential lung nodules.	CT	Siemens Healthcare K.K.	Siemens Healthcare GmbH	Standalone + Physician-assisted test
14	30200BZX00184000	2nd ed., Sep 23, 2020	Analyzes chest CT scans for findings associated with COVID-19 pneumonia, providing reference information.	CT	CES Descartes, Inc.	Beijing Infervision Technology Co.,Ltd.	Standalone test
15	30300BZX00145000	2nd ed., Feb 2024	Analyzes chest CT scans to show the likelihood of findings seen in COVID-19 pneumonia.	CT	FUJIFILM Corporation	FUJIFILM Corporation	Standalone test
16	30200BZX00212000	1st ed., Jul 2020	Analyzes chest CT scans to indicate the likelihood of findings related to COVID-19 pneumonia.	CT	MIC Medical Corporation	Alibaba Damo (Hangzhou) Technology Co., Ltd	Standalone test
17	30400BZX00123000	1st ed., Jul 2022	Analyzes chest CT scans to help identify findings suggestive of COVID-19 pneumonia.	CT	Canon Medical Systems Corporation	Canon Medical Systems Corporation	Standalone test
28	30600BZX00155000	1st ed., Jul 29, 2024	Analyzes fetal heart ultrasound videos to help doctors assess heart structures and check for abnormalities.	US	Fujitsu Japan Limited	Fujitsu Japan Limited	Standalone + Physician-assisted test
18	30300BZX00350000	5th ed., May 31, 2024	Analyzes chest CT images to highlight potential findings of COVID-19 pneumonia for doctors.	CT	Fujitsu Japan Limited	Fujitsu Japan Limited	Standalone test
21	30600BZX00098000	1st ed., May 2024	Analyzes panoramic dental X-rays to help dentists evaluate jawbone thickness and shape.	CR	MEDIA Co., Ltd.	MEDIA Co., Ltd.	Standalone test
31	30100BZX00142000	7th ed., Jun 2025	Analyzes head MRA scans to help doctors detect potential brain aneurysms.	MR	LPIXEL Inc.	LPIXEL Inc.	Standalone + Physician-assisted test
22	30400BZX00285000	4th ed., May 2024	Analyzes chest X-rays to help detect and mark potential abnormal shadows, such as nodules or infiltrates.	CR	LPIXEL Inc.	LPIXEL Inc.	Standalone + Physician-assisted test
23	30200BZX00269000	2nd ed., Jun 2024	Analyzes chest X-rays to help doctors find and mark potential lung nodules.	CR	LPIXEL Inc.	LPIXEL Inc.	Standalone + Physician-assisted test
24	30300BZX00271000	5th ed., Oct 2024	Analyzes chest X-rays to help detect patterns similar to nodules, masses, and infiltrates.	CR	Konica Minolta, Inc.	Konica Minolta, Inc.	Standalone + Physician-assisted test
25	30300BZX00188000	10th ed., Mar 2025	Analyzes chest X-rays to detect potential lung nodules, infiltrates, and pneumothorax, showing results as a heatmap.	CR	FUJIFILM Corporation	FUJIFILM Corporation	Standalone + Physician-assisted test
26	30300BZX00339000	1st ed., Jan 2022	Analyzes chest X-rays to provide a confidence score for findings related to infectious pneumonia.	CR	Doctor-Net Inc.	JLK Inc.	Standalone test
27	30500BZX00262000	1st ed., Dec 2023	Analyzes chest X-rays to help detect findings suggestive of fibrotic interstitial lung disease.	CR	COSMOTEC Co., Ltd.	COSMOTEC Co., Ltd.	Standalone + Physician-assisted test
29	30200BZX00379000	1st ed., Nov 24, 2020	Analyzes breast ultrasound images in real-time to help identify and mark potential lesions.	US	CES Descartes, Inc.	TaiHao Medical Inc. (Taiwan)	Standalone + Physician-assisted test
30	30600BZX00086000	1st ed., Mar 2025	Analyzes breast ultrasound images to help detect suspicious lesions and assess the need for further examination.	US	Smart Opinion, Inc.	Smart Opinion, Inc.	Standalone + Physician-assisted test
19	30200BZX00150000	10th ed., Oct 2024	Analyzes chest CT scans to help doctors detect and mark potential lung nodules.	CT	FUJIFILM Corporation	FUJIFILM Corporation	Standalone + Physician-assisted test
32	30100BZX00263000	4th ed., Nov 2022	Searches a hospital’s database for past cases with imaging findings similar to a user-selected area.	Undefined (likely CT)	FUJIFILM Corporation	FUJIFILM Corporation	Standalone test
20	30300BZX00244000	9th ed., Oct 2024	Analyzes chest CT scans to help doctors find and mark potential rib fractures.	CT	FUJIFILM Corporation	FUJIFILM Corporation	Standalone + Physician-assisted test

Table 1 shows a summary of each product. The 20 included radiology AI products demonstrated diverse applications across imaging modalities: CT imaging dominated with eight products [13–20] (40.0%); computed radiography (CR) accounted for seven products [21–27] (35.0%); ultrasound (US) for three products [28–30] (15.0%); MRI for one product [31] (5.0%); and one product (5.0%) had no imaging modality explicitly specified (likely CT) [32]. Five products (25.0%) were specifically developed for COVID-19-related imaging findings [14–18].

### Transparency in clinical setting reporting

**Fig. 2:**
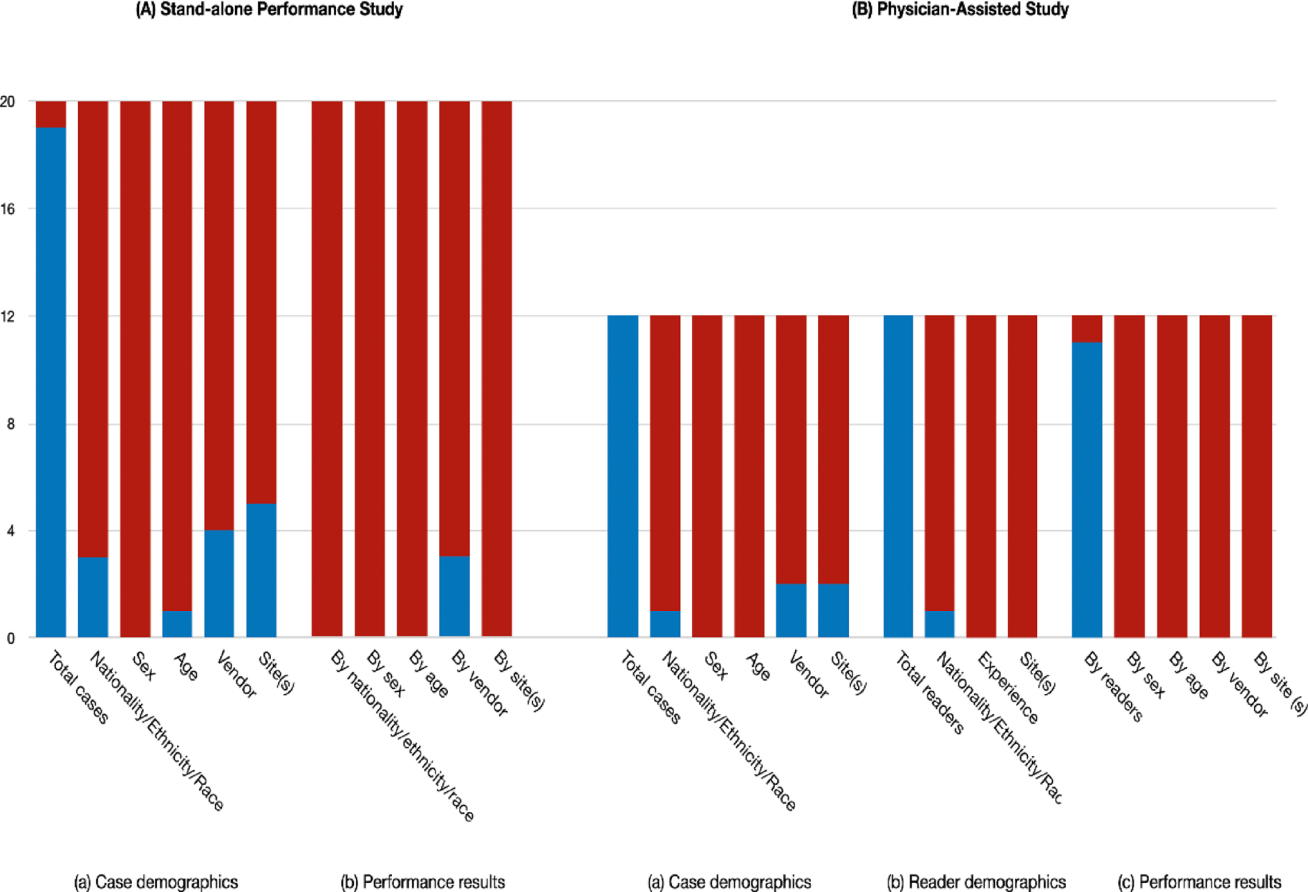
Data availability within each product. Availability of demographic and performance data for the 20 included products. The bar charts show the number of products with data publicly available in regulatory documents (blue) or not reported (red). Data availability is shown separately for **A** Stand-alone performance studies and **B** Physician-assisted studies. Categories assessed include Case demographics, Reader demographics, and Performance results stratified by various factors

Analysis of data availability revealed significant gaps in the reporting of clinical study characteristics (Fig. 2) (Table 2). In standalone performance studies, none of the products (20/20, 100%) provided complete demographic information—including nationality/ethnicity/race, sex, and age. Most products (16/20, 80%) reported only the number of cases without any demographic breakdown.

**Table 2:** Stand-alone performance study

Approved ID	Case demographics							Performance results					
	Total cases (n)	Positive cases (n)	Nationality/Ethnicity/Race	Sex	Age	Vendor	Site(s)	Main	By nationality/ethnicity/race	By sex	By age	By vendor	By site(s)
30200BZX00202000	232 cases	–	USA	–	–	Siemens, GE, Philips, Canon	5 US institutions and 2 public databases	Lung lobe: Sensitivity 85.1%, Specificity 75.8% Lung nodule: Sensitivity 67.7%, False positives per patient (FPP) 1.784	–	–	–	Siemens: Sensitivity 69%, FPP 2.9 GE: Sensitivity 65%, FPP 2.0 Philips: Sensitivity 67%, FPP 1.5 Canon: Sensitivity 69%, FPP 1.3	–
30200BZX00184000	190 cases	COVID PCR+: 83 cases	Japan	–	–	–	Institution A and B	Sensitivity 77.1%; Specificity 90.7%	–	–	–	–	–
30300BZX00145000	217 cases	COVID PCR+: 112 cases	–	–	–	–	–	Medium or higher confidence: Sensitivity 87.5%, Specificity 37.1%	–	–	–	–	–
30200BZX00212000	704 cases	COVID PCR+: 327 cases	–	–	–	–	–	Sensitivity 89.6%, Specificity 37.1%	–	–	–	–	–
30400BZX00123000	174 cases	COVID PCR+: 87 cases	–	–	–	–	–	Sensitivity 88.5%, Specificity 47.1%	–	–	–	–	–
30600BZX00155000	285 images from 95 videos (72 cases)	–	–	–	Fetus	–	–	Sensitivity: 93.5%; Specificity: 95.9%	–	–	–	–	–
30300BZX00350000	201 cases	COVID PCR+: 120 cases	–	–	–	–	–	“Mid” or higher: Sensitivity 85.0%, Specificity 45.7%; “High”: Sensitivity 55.0%, Specificity 81.5%	–	–	–	–	–
30600BZX00098000	194 cases	NA	–	–	–	–	Multiple	Agreement rate with Gold Standard: 79.9%	–	–	–	–	–
30100BZX00142000	–	–	–	–	–	–	–	Sensitivity 96.5%; False positives per case 1.16	–	–	–	–	–
30400BZX00285000	354 cases	88 cases with findings	–	–	–	KONICA, Fuji Film Medical	–	Per-finding: Sensitivity 64.3%, PPV 56.7%, Per-case: Specificity 90.6%, NPV 90.9%	–	–	–	KONICA: Sensitivity 61.4%, Specificity 89.7% Fuji: Sensitivity 67.3%, Specificity 91.5%	–
30200BZX00269000	320 cases	67 cases with 76 nodules	–	–	–	KONICA, Philips, Fuji Film Medical	–	Sensitivity 78.4%; Positive Predictive Value 77.3%	–	–	–	KONICA: Sensitivity 80.6%, Specificity 93.9% Philips: Sensitivity 75.9%, Specificity 90.9% Fuji: Sensitivity 77.8%, Specificity 97.6%	–
30300BZX00271000	409 images	103 nodules/masses, 93 infiltrates	–	–	–	–	–	Sensitivity (nodules/masses) 84%; Sensitivity (infiltrates) 85%; Specificity 88%; False positives per image 0.25	–	–	–	–	–
30300BZX00188000	267 images	72 abnormal images	–	–	–	–	–	Results provided per threshold. E.g., at threshold ≥ 30: Sensitivity 90.3%, Specificity 92.8%	–	–	–	–	–
30300BZX00339000	297 images	106 abnormal images	–	–	–	–	–	Confidence ≥ 30%: Sensitivity 98.1%, Specificity 36.6%; Confidence ≥ 65%: Sensitivity 53.8%, Specificity 91.6%	–	–	–	–	–
30500BZX00262000	1372 images	87 fibrotic ILD positive	–	–	–	–	5 institutions	Sensitivity 87.4%; Specificity 89.8%	–	–	–	–	–
30200BZX00379000	230 cases	–	Taiwan	–	–	–	Single	Per-lesion sensitivity 95.5%; FPs per case: 28.1 frames; Per-case sensitivity 100.0% and specificity 2.2%	–	–	–	–	–
30600BZX00086000	50 images	–	–	–	–	–	–	Accuracy 80.0%; Sensitivity 100.0%; Specificity 73.0%	–	–	–	–	–
30200BZX00150000	36 cases	28 abnormal images	–	–	–	–	–	Sensitivity 78.4%	–	–	–	–	–
30100BZX00263000	30 cases	–	–	–	–	–	–	Verified that the success criterion (70%) was met.	–	–	–	–	–
30300BZX00244000	64 images	32 abnormal images	–	–	–	Canon, Siemens, Philips	–	Sensitivity 89.3%, 1.61 false positives per case.	–	–	–	–	–

Physician-assisted studies showed similarly limited transparency in reader characteristics (Fig. 2) (Table 3). Among the 12 products that included physician-assisted validation, most studies (11/12, 92%) detailed reader experience levels, but only one (1/12, 8%) reported reader nationality or practice location.

**Table 3:** Physician-assisted study

Approved ID	Case demographics							Reader demographics				Performance results					
	Total cases (n)	Positive cases (n)	Nationality/Ethnicity/Race	Sex	Age	Vendor	Site(s)	Total readers (n)	Nationality/Race	Experience	Site(s)	Main	By readers	By sex	By age	By vendor	By site (s)
30200BZX00202000	232 cases	–	US	–	–	Siemens, GE, Philips, Canon	Multi	20	US	Radiologists	–	AUC without AI: 0.744, with AI: 0.785–0.788 (*p* < 0.001)	–	–	–	–	–
30600BZX00155000	60 videos	20 videos	–	–	–	–	–	44	–	30 specialists 14 residents	–	Sensitivity without AI: 73.6%, with AI: 78.4% (*p* = 0.005) Specificity without AI: 79.1%, with AI: 86.5% (*p* < 0.001)	Residents: Sensitivity 63.2% to 68.6% Specificity 79.8% to 89.3% Specialist: Sensitivity 78.5% to 83.0% Specificity 78.8% to 85.3%	–	–	–	–
30100BZX00142000	200 cases	50 aneurysms	–	–	–	–	–	20	–	10 radiologists 10 neurosurgeons	–	FOM without AI: 0.7168, with AI: 0.7514 (*p* < 0.001)	Radiologists < 5 years exp: 0.6470 to 0.6972 Radiologists ≧ 5 years exp: 0.7990 to 0.8218 Neurosurgeon < 6 years exp: 0.6445 to 0.6813 Neurosurgeon ≧ 6 years exp: 0.8096 to 0.8362	–	–	–	–
30400BZX00285000	354 cases	88 cases with findings	–	–	–	–	–	10	–	3 radiologists 7 non-specialists	–	FOM without AI: 0.714, with AI: 0.771 (*p* < 0.001)	Radiologists: 0.713 to 0.772 Non-specialists: 0.714 to 0.771	–	–	–	–
30200BZX00269000	320 cases	67 cases with 76 nodules	–	–	–	–	–	18	–	9 radiologists 9 non-specialists	–	AUC without AI: 0.7088, with AI: 0.7688 (*p* < 0.001)	Radiologists: 0.7173 to 0.7683 Non-specialists: 0.7002 to 0.7693	–	–	–	–
30300BZX00271000	200 cases	60 with findings	–	–	–	–	–	12	–	4 radiologists 8 internal physicians	–	FOM without AI: 0.756, with AI: 0.878 (*p* < 0.025)	Physicians ≦ 5 years exp: 0.751 to 0.870 Physicians ≧ 6 years exp: 0.761 to 0.887	–	–	–	–
30300BZX00188000	267 images	72 abnormal images	–	–	–	–	–	10	–	3 specialists 7 non-specialists	–	FOM without AI: 0.771, with AI: 0.839 (*p* < 0.05)	Specialists: 0.837 to 0.862 Non-specialists ≧ 10 years exp: 0.724 to 0.829 Non-specialists < 5 years exp: 0.756 to 0.829	–	–	–	–
30500BZX00262000	85 images	19 fibrotic ILD	–	–	–	–	Multi	25	–	5 specialists 20 non-specialists	–	AUC without AI: 0.8079, with AI 0.8457 (*p* < 0.0001)	Specialists: 0.8917 to 0.8969 (*p* = 0.1181) Non-specialists: 0.7876 to 0.8313 (*p* = 0.0002)	–	–	–	–
30200BZX00379000	51 cases		–	–	–	–	–	18	–	6 specialists 6 junior doctors 6 medical technologists	–	FOM without AI: 0.6304, with AI: 0.7726 (*p* < 0.0001)	Specialists: 0.6245 to 0.7430 Junior doctors: 0.5837 to 0.7706 Medical technologist: 0.6830 to 0.8042	–	–	–	–
30600BZX00086000	50 images		–	–	–	–	–	24	–	4 specialists 20 non-specialists	–	Accuracy without AI: 69.3%, with AI: 73.1% (*p* = 0.005)	Specialists: 75.5% to 76.5% Experienced non-specialists: 77.1% to 79.6% Unexperienced non-specialists: 64.7% to 69.2%	–	–	–	–
30200BZX00150000	36 images	28 abnormal	–	–	–	–	–	10	–	5 specialists 5 residents	–	FOM without AI: 0.638, with AI: 0.674 (*p* < 0.05)	Specialists: 0.668 to 0.702 Residents: 0.609 to 0.646	–	–	–	–
30300BZX00244000	64 images	32 abnormal images	–	–	–	Canon, Siemens, Philips	–	10	–	5 specialists 5 residents	–	FOM without AI: 0.782, with AI: 0.820 (*p* < 0.01)	Specialists: 0.765 to 0.821 Residents: 0.799 to 0.820	–	–	–	–

### Clinical performance metrics

Standalone performance studies demonstrated wide variability in reported metrics (Table 2). Differences in experimental design, reported outcomes, and chosen evaluation metrics meant that comparability across products was not necessarily high. Sensitivity values ranged from 55.0% to 100%, with COVID-19 detection applications generally showing variable performance depending on confidence thresholds. Specificity showed even greater variability, ranging from 2.2% to 97.6%, with several products reporting specificity below 50%.

Physician-assisted studies consistently demonstrated performance improvements with AI assistance (Table 3). Twelve products reporting physician-assisted validation showed statistically significant improvements in primary performance metrics. The magnitude of improvement varied considerably across studies. Notably, less experienced readers showed greater performance gains, with one study reporting AUC improvement from 0.6470 to 0.6972 for junior neurosurgeons compared to 0.7990 to 0.8218 for experienced radiologists [31].

### Subgroup performance analysis

Critical gaps were identified in subgroup performance reporting. No products provided performance metrics stratified by patient age, sex, or ethnicity, despite the known impact of demographic factors on AI performance. Only 3 products (15.0%) reported vendor-specific performance differences, revealing substantial variations. For example, one lung nodule detection system showed sensitivity ranging from 65% on GE equipment to 69% on Canon systems, with false positive rates varying from 1.3 to 2.0 per patient across vendors. Site-specific performance data were absent from all package inserts, preventing assessment of generalization across different clinical settings.

## Discussion

This scoping review reveals fundamental gaps in the transparency of clinical evidence for PMDA-approved AI-based radiology SaMD, with significant implications for clinical implementation and patient safety. The finding that none of the products provide complete demographic information and none offer subgroup-stratified performance metrics represents a critical barrier to evidence-based adoption of these technologies in Japanese healthcare settings.

### Implications for clinical implementation

The wide variability in performance metrics underscores the importance of understanding the specific clinical context and validation conditions for each AI system [4]. Without detailed demographic information or site-specific performance data, clinicians cannot adequately assess whether published performance metrics will translate to their specific patient populations. This uncertainty may lead to either inappropriate rejection of beneficial technologies or, conversely, over-reliance on systems that may not perform as expected in local contexts [9].

Of the twenty AI‑based radiology products, twelve included physician‑assisted studies. The consistent performance improvements demonstrated in physician-assisted studies suggest that AI integration can enhance diagnostic accuracy across experience levels. However, the greater gains observed among less experienced readers raise important questions about training requirements and the potential for AI to either reduce or exacerbate expertise-related disparities in diagnostic performance [33]. No physician‑assisted study reported performance results other than by-reader analyses, a notable omission given the heterogeneity of patient populations, imaging protocols, and clinical workflows across Japanese healthcare institutions.

The five COVID-19 detection applications warrant separate analysis given their rapid development and deployment timeline, reflecting the pandemic’s influence on AI development priorities. All of these products were approved based solely on standalone performance studies without reader studies. Consequently, each product received conditional approval requiring that appropriate post-marketing studies be conducted to evaluate the product’s performance and clinical utility. These products showed variable performance metrics, with sensitivity ranging from 55.0% to 89.6% (depending on confidence thresholds) and specificity from 37.1 to 90.7%. None provided information about variant-specific performance or temporal validation despite the evolving nature of COVID-19 imaging findings.

### Regulatory evolution and international comparisons

Japan’s PMDA has been progressive in establishing approval pathways for AI-based medical devices, yet our findings suggest that documentation requirements have not evolved to match the unique characteristics of AI systems [34]. A recent study by Shick et al. analyzing 691 FDA-cleared AI devices found that 95.5% of regulatory summaries lacked demographic data, mirroring the opacity we observed in Japan [8]. However, the regulatory response differs: the US FDA has actively released “Transparency for Machine Learning-Enabled Medical Devices: Guiding Principles” to address these gaps [5]. Furthermore, the European Union’s upcoming AI Act mandates strict technical documentation for high-risk AI. In contrast, Japan’s current framework prioritizes efficiency, potentially at the cost of mandated public disclosure. It is also crucial to address the role of third-party certification (via Registered Certification Bodies) in Japan. Although our study focused exclusively on PMDA-approved products and excluded third-party certified devices to ensure data homogeneity and focus on products subject to direct PMDA review, this certification pathway is widely utilized for Class II (Controlled) medical devices that meet established standards. This system effectively accelerates the market entry of ‘me-too’ devices and ensures rapid clinical adoption.

The contrast between Japan’s advanced regulatory framework for AI approval and the limited transparency requirements for clinical evidence creates a paradox. While the PMDA’s efficiency in approving AI products has positioned Japan in the top tier of medical AI adoption, the lack of detailed performance information may ultimately hinder successful clinical integration and potentially compromise patient safety [3].

### The role of real-world performance monitoring

The gaps identified in pre-market clinical evidence underscore the critical importance of post-market surveillance and real-world performance monitoring for AI-based medical devices. Unlike traditional medical devices, AI systems can exhibit performance drift over time due to changes in patient populations, imaging protocols, or clinical practices [35]. Current package insert requirements do not address this dynamic nature of AI performance, nor do they provide frameworks for ongoing validation.

This situation creates opportunities for specialized organizations that can bridge the gap between regulatory compliance and clinical implementation needs. Companies with expertise in Japan’s Next Generation Medical Infrastructure Act and Personal Information Protection Act are uniquely positioned to facilitate privacy-preserving, multi-institutional validation studies that can provide the real-world evidence lacking in current regulatory documentation. Such organizations can establish standardized frameworks for continuous performance monitoring, ensuring that AI systems maintain their expected performance across diverse clinical settings while complying with Japan’s strict data privacy regulations.

### Recommendations for enhanced transparency

Based on our findings, we propose several recommendations for enhancing transparency in AI-based radiology SaMD documentation. First, package inserts should include mandatory reporting of demographic characteristics for both training and validation datasets, with performance metrics stratified by key demographic variables. Second, vendor-specific and site-specific performance data should be required when technically feasible, acknowledging the impact of imaging equipment and clinical workflows on AI performance.

Third, for physician-assisted studies, detailed reader characteristics including specialization, experience levels, and geographic distribution should be standard requirements. This information is essential for healthcare institutions to assess the training and implementation requirements for their specific workforce. Fourth, products should include clear statements about the reference standards used for validation, particularly important for conditions like COVID-19 where diagnostic criteria have evolved over time.

### Industry innovation and future directions

The current regulatory landscape creates both challenges and opportunities for innovation in medical AI validation and implementation. Organizations specializing in medical data governance and AI validation can play a crucial role in establishing industry standards that exceed minimum regulatory requirements. By leveraging frameworks such as the Next Generation Medical Infrastructure Act, these organizations can create secure, privacy-preserving environments for continuous AI validation across multiple institutions.

Furthermore, the development of standardized reporting templates and performance monitoring dashboards could significantly enhance transparency while reducing the burden on individual manufacturers. Industry-led initiatives for voluntary transparency standards, potentially coordinated through professional societies or industry associations, could accelerate the evolution of regulatory requirements while immediately benefiting clinical users.

### Limitations

First, our analysis was limited to publicly available package inserts, focusing strictly on “regulatory transparency.” We acknowledge that additional technical details may exist in manufacturer white papers, PMDA review reports, or academic publications (contributing to “overall transparency”); however, such information could not be captured in this study. Second, we lacked access to the raw clinical‑study data underlying package‑insert claims, preventing independent verification of performance metrics and deeper assessment of study quality. Third, this review focused exclusively on PMDA-approved products; third-party-certified AI-based SaMD products were not evaluated. As discussed, these products play a significant role in the Japanese market, and their transparency standards warrant future investigation.

## Conclusion

This scoping review of PMDA-approved AI-based radiology SaMD reveals significant gaps in the transparency and completeness of clinical evidence available to healthcare providers. The absence of demographic stratification, vendor-specific performance data, and detailed validation conditions creates uncertainty for clinicians and healthcare institutions seeking to adopt these technologies. Addressing these gaps requires coordinated efforts from regulators, manufacturers, and specialized organizations with expertise in medical data governance and AI validation. Enhanced transparency standards, coupled with robust frameworks for continuous real‑world performance monitoring, are essential to unlock the full potential of AI in Japanese radiology practice while safeguarding clinical effectiveness and patient safety.

## Data Availability

The data analyzed in this study were extracted from publicly available sources and are generally accessible. All analytical results are summarized in the tables within the manuscript.
